# Estimating Sensitivity of Laboratory Testing for Influenza in Canada through Modelling

**DOI:** 10.1371/journal.pone.0006681

**Published:** 2009-08-18

**Authors:** Dena L. Schanzer, Michael J. Garner, Todd F. Hatchette, Joanne M. Langley, Samina Aziz, Theresa W. S. Tam

**Affiliations:** 1 Infectious Disease and Emergency Preparedness Branch, Public Health Agency of Canada, Ottawa, Ontario, Canada; 2 Canadian Centre for Vaccinology, QEII Health Sciences Centre and Faculty of Medicine, Dalhousie University, Halifax, Nova Scotia, Canada; 3 Canadian Centre for Vaccinology, IWK Health Centre and Faculty of Medicine, Dalhousie University, Halifax, Nova Scotia, Canada; Singapore Immunology Network, Singapore

## Abstract

**Background:**

The weekly proportion of laboratory tests that are positive for influenza is used in public health surveillance systems to identify periods of influenza activity. We aimed to estimate the sensitivity of influenza testing in Canada based on results of a national respiratory virus surveillance system.

**Methods and Findings:**

The weekly number of influenza-negative tests from 1999 to 2006 was modelled as a function of laboratory-confirmed positive tests for influenza, respiratory syncytial virus (RSV), adenovirus and parainfluenza viruses, seasonality, and trend using Poisson regression. Sensitivity was calculated as the number of influenza positive tests divided by the number of influenza positive tests plus the model-estimated number of false negative tests. The sensitivity of influenza testing was estimated to be 33% (95%CI 32–34%), varying from 30–40% depending on the season and region.

**Conclusions:**

The estimated sensitivity of influenza tests reported to this national laboratory surveillance system is considerably less than reported test characteristics for most laboratory tests. A number of factors may explain this difference, including sample quality and specimen procurement issues as well as test characteristics. Improved diagnosis would permit better estimation of the burden of influenza.

## Introduction

Although influenza virus infection is associated with considerable morbidity and mortality[1]–[3], laboratory confirmation of clinical illness is the exception rather than the rule. Clinicians do not routinely seek laboratory confirmation for several reasons: diagnosis will often not alter patient management, a paucity of real-time, accurate, inexpensive testing methods [4] and because influenza is not recognized as the etiology of the clinical presentation[5]. Accurate diagnosis of influenza-like illness, however, could improve clinical care through reduced use of antibiotics and ancillary testing, and more appropriate use of antiviral therapy [6]. Although rapid influenza tests such as point-of-care tests are purported to generate results in a timely fashion to influence clinical care, the performance characteristics of the currently available tests are sub-optimal [7]. New technologies with improved sensitivity such as reverse-transcriptase polymerase chain reaction (RT-PCR) [8] as well as the use of more effective collection systems such as the flocked nasopharyngeal swab compared to traditional rayon wound swabs, and the recommendation to collect more ideal specimens, such as nasopharyngeal swabs rather than throat swabs are likely to improve diagnostic sensitivity [9]–[12]. The performance characteristics of currently available tests for influenza vary considerably and the overall sensitivities of these tests when used in routine practice are also dependent on the type of specimen collected, the age of the patient and point in their illness in which they are sampled [4], [9], [13]–[15].

We sought to estimate the sensitivity of influenza testing based on results of a national respiratory virus surveillance system using a model-based method [1], [2], [16]–[18].

## Methods

### Sources of data

Weekly respiratory virus identifications from September 1999 to August 2006 were obtained from the Respiratory Virus Detection Surveillance System (RVDSS), Public Health Agency of Canada [19], [20]. The RVDSS collects, collates, and reports weekly data from participating laboratories on the number of tests performed and the number of specimens confirmed positive for influenza, respiratory syncytial virus (RSV), para-influenza virus (PIV), and adenovirus. Specimens are generally submitted to laboratories by clinicians in the course of clinical care, and by clinicians participating in one of our national influenza surveillance programs, (*FluWatch* [20]). Indicators of influenza activity are reported year round on a weekly basis to the *FluWatch* program. The RVDSS is supplemented by case reports of influenza positive cases [19], [21]. From the case reports, influenza A was confirmed in all age groups and sporadic cases were confirmed in the off-season months of June through September. Infants and children under the age of 5 years accounted for 25% of the influenza A positive tests, and persons over the age 65 years another 35%. Unfortunately, *FluWatch* surveillance data does not provide the total number of tests by age. Testing practices are known to be varied [22], [23]. The predominant testing methods used for influenza detection varied considerably by province or laboratory and over time. For the 2005/06 season a survey of laboratory techniques in current use indicated that culture accounted for 44% of the diagnostic tests with RT-PCR, rapid antigen tests and direct fluorescent-antibody assay (DFA) accounting for 21%, 19%, and 16% respectively[23].

### Statistical Analysis

The weekly number of tests negative for influenza was modelled, using Poisson regression, as a function of viral identifications for influenza, RSV, adenovirus and PIV as well as a baseline consisting of seasonality, trend and holiday variables. The estimated baseline implicitly accounts for influenza tests on specimens taken from patients with respiratory infections due to respiratory pathogens other than the four viruses captured in the RVDSS, as long as both the testing behaviour of clinicians and respiratory illnesses caused by other respiratory pathogens follow a consistent seasonal pattern as prescribed by the model (see below, parameters *β*_1_ to *β*_4_).

The Poisson regression model with a linear link function was estimated using SAS [24] PROC GENMOD:
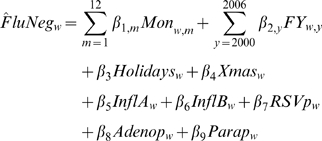
where 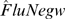 is the predicted number of negative tests for influenza for week *w*; *Mon_w,m_* an indicator variable for each month; *FY*_w,y_ an indicator variable for the influenza season (year running from September to August); *Holidays*_w_ and *Xmas*_w_ variables indicating holidays; *InflA_w_, InflB_w,_ RSVp_w_, Adenop_w_, Parap_w_* the weekly number of tests confirmed positive for influenza A and B, RSV, adenovirus and para-influenza virus respectively. A regression model approach facilitates the simultaneous estimation of the effects of influenza activity on the number of influenza-negative tests while controlling for other factors. The model was further stratified by influenza season by including separate parameters for each season (β_5y_ rather than β_5_).

Coefficients *β*_5_ to *β*_9_ are multipliers. The weekly number of influenza negative tests estimated to be falsely negative is given by *β*_5_ *InflA*_w_+*β*_6_ *InflB*_w_. The weekly number of influenza negative tests attributed to RSV is given by *β*_7_ *RSVp*_w._, and similarly for adenovirus and PIV. For each positive influenza A test, an additional *β*_5_ tests above baseline were performed and found to be negative. By specifying a linear link, a value of 0.33, say, for coefficient *β*_5_, means that for every test for which influenza A was confirmed, 0.33 additional tests, on average, were performed on truly influenza A positive specimens and found to be negative – which corresponds to a sensitivity of 75%.

Sensitivity was calculated as the number of influenza positive tests divided by the number of influenza positive tests plus the model-estimated number of false negative tests, or equivalently, the estimates of sensitivity for influenza A and B are given by 1/(1+*β*_5_) and 1/(1+*β*_6_) respectively. The false negative rate is 1 minus sensitivity. While the null value for *β*_5_ is zero, which indicates no statistical association between the number of influenza positive tests and the number of influenza negative tests, the corresponding null value for sensitivity is 1.

For each test confirmed positive for RSV, on average β_7_ tests were performed for influenza and found to be negative for influenza. These β_7_ tests are attributed to an RSV infection, however the number of influenza-negative tests that actually tested positive for RSV is unknown. If all specimens had been tested for the same viruses (panel tests), 1/*β*_7_ would correspond to the sensitivity for RSV testing, and the sensitivity for adenovirus and PIV given by 1/*β*_8_ and 1/*β*_9_ respectively. Some laboratories are known to test for viruses sequentially [22], and so 1/*β*_7_ - 1/*β*_9_ were not interpreted as estimates of the sensitivity for other viruses. Sequential testing may occur if a rapid test for influenza is negative and the laboratory then performs PCR or culture testing. Similarly in young children with a respiratory illness in the winter, rapid tests for RSV infection may be performed first, and only specimens with negative results submitted for subsequent testing for influenza or other respiratory viruses [25]. By contrast, many laboratories conduct panel tests for multiple viruses for ease of handling, decreased patient sampling, and recognition that co-infection can occur. Either form of sequential testing would not bias the estimate of sensitivity applicable to test results reported to RVDSS, though significant use of rapid antigen tests in the laboratories reporting to RVDSS would reduce the overall sensitivity. As a single specimen may undergo multiple tests, the false-negative rate applicable to a specimen that has undergone multiple tests would be expected to be much lower than the system average for individual tests. Parameters *β*_1._ to *β*_4_ account for trends and the seasonality of truly negative specimens (patients presenting with other acute respiratory infections).

## Results

**Figure 1:**
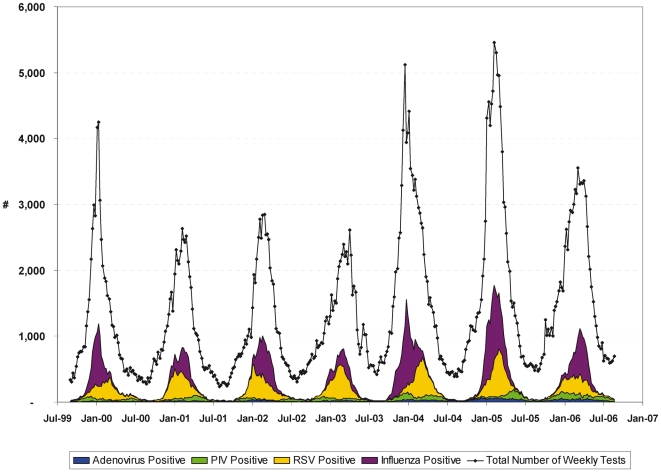
RVDSS viral identifications. Weekly number of specimens tested for influenza is shown with the number of tests confirmed positive for influenza (A and B), adenovirus, parainfluenza virus, and RSV. Data is presented ignoring co-infection and sequential testing, so the white area under the curve, which corresponds to 75% of tests, represents the minimum number of specimens that were negative for all 4 viruses.

Over 50,000 tests for influenza were reported to the RVDSS each year, peaking in 2004/05 at 101,000. Overall 10% of the influenza tests were positive for influenza, ranging from 4% to 13% depending on the season. The proportion positive for RSV, parainfluenza and adenovirus averaged 9%, 3% and 2% respectively. As seen in Figure 1, no virus was identified in 75% of specimens submitted for testing (white area under the curve). Even for the winter months of December through April, one of these 4 viruses was identified on average in no more than 30% of the specimens. The strong and consistent synchronization of negative tests with influenza positive tests, as seen in Figure 1, is suggestive that false negative results contributed to the large number of negative tests during periods of influenza activity.

The sensitivity for influenza A testing averaged 33.7% (with model-estimated 95% confidence intervals of 33.3–34.1) for the 1999/2000–2005/06 period. Influenza B testing had a similar estimated sensitivity at 34.7 (95% CI 33.4–36.1). Estimated sensitivities varied somewhat from season to season, generally ranging from 30%–40% (Table 1), and provincial level estimates, as well, were within a similar range. Stratifying by province or season produced similar estimates for the sensitivity of influenza A testing: 32% (95% CI 30–34) and 36% (95% CI 33–41) respectively. Estimates of sensitivity based on test results reported to the RVDSS for individual laboratories with sufficient data to fit the model showed significant variation, with estimates of sensitivity ranging from 25–65%. As expected, laboratories using primarily rapid antigen tests had lower estimated sensitivities, and laboratories that used PCR methods had higher sensitivity estimates. However, information on testing procedures is limited primarily to the 2005/06 survey. As well, additional irregularities were noticed in the laboratory data and not all laboratories provided sufficient data to fit the model.

**Table 1:** Model Estimates of Sensitivity for Influenza A Testing as Reported to the RVDSS, by Influenza Season.

Season	Sensitivity	95% CI
1999/00	34%	(32%–38%)
2000/01	80% (ns)	(39%–100%)
2001/02	48%	(41%–56%)
2002/03	n/a	
2003/04	35%	(32%–37%)
2004/05	34%	(32%–37%)
2005/06	35%	(30%–44%)
**Weighted Average**	36%	(33%–41%)

Note: Estimates of sensitivity by influenza season were obtained by estimating separate β_5,y_ parameters, one for each season. Noting that the null value for sensitivity is 100%, as 100% sensitivity implies that there should no association between the number of influenza negative and influenza positive tests, the season specific estimates appear to be reasonably consistent. Season specific differences in the estimated sensitivity may be due to irregular reporting and the tendency of data irregularities to bias the model parameters β_5,y_ towards the null, or sensitivity towards 1. A value of 100% for sensitivity implies that there is no association between the number of influenza negative and influenza positive tests. The 2000/01 and 2002/03 season estimates (both H1N1/B seasons) were uninformative. This lack of statistical significance and wide confidence intervals were attributed to the relatively small number of influenza A positive specimens in these two H1N1 seasons. A shift in influenza A confirmations towards younger ages was noted during the H1N1 seasons. Testing a larger proportion of children may result in an improvement in the overall test sensitivity.

ns: Not statistically significant. The null value for sensitivity is 100%. With 100% sensitivity no association between the number of influenza negative and influenza positive tests would be expected.

n/a: Not available. Estimate was out of range and not statistically significant.

**Figure 2:**
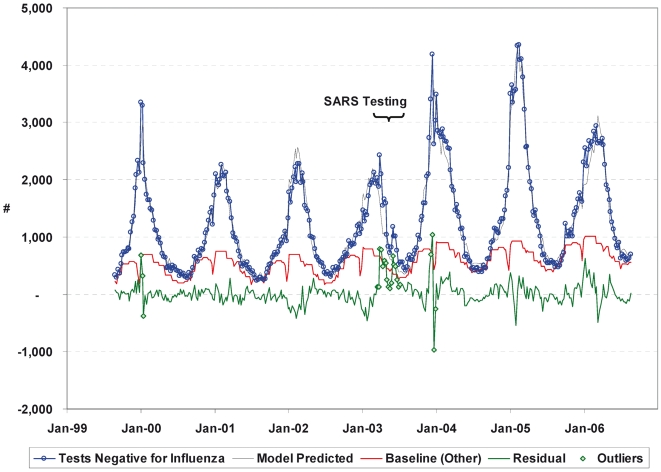
Model predicted number of tests negative for influenza. The weekly number of influenza tests not confirmed positive for influenza was modelled as a function of viral identifications for influenza, RSV, adenovirus and parainfluenza, seasonality, and trend using Poisson regression. Identified outliers, corresponding to periods with irregular testing were excluded from the model. The baseline accounts for routine tests in the hypothetical absence of influenza, RVS, adenovirus and parainfluenza activity.

Figure 2 illustrates a good model fit where the weekly number of influenza negative tests is well explained by the model covariates, with a few exceptions. Firstly, it is evident that additional specimens were tested during the SARS period, as indicated by the period where the number of weekly influenza negative tests exceeded the expected number, or equivalently, a period of successive positive residuals. Residuals typically capture random variation; hence represent tests that can not be allocated based on the specified model. In addition to the SARS period, testing appears to have been elevated for a number of weeks in January 2000 during the peak of the 1999/2000 A/Sydney/05/97 (H3N2) season in which respiratory admissions were unusually elevated [26], [27], and in December 2003, when an elevated risk of paediatric deaths associated with the A/Fujian/411/02 (H3N2) strain [28] was identified in the US. As these periods corresponded to a period of heightened public awareness due to severe influenza outbreaks, parameter estimation was repeated without these data points. Exclusion of these data points did not alter the sensitivity estimate for influenza.

**Figure 3:**
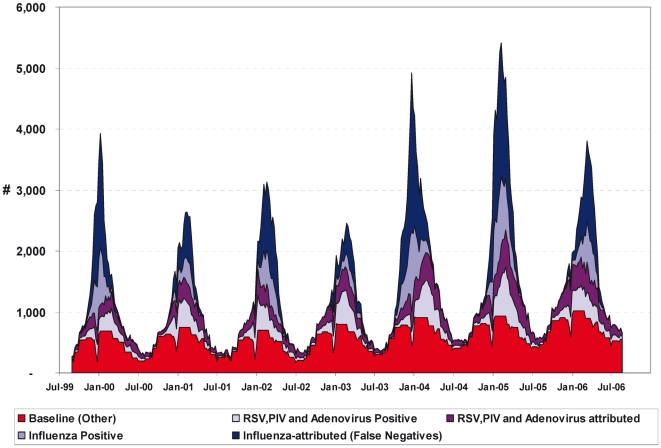
Attribution of Specimens Tested for Influenza and Reported to the RVDSS, Canada. The modelled attribution of the weekly number of specimens tested for influenza to influenza (A and B), and adenovirus, parainfluenza virus, and RSV combined is shown along with the numbers confirmed positive. The total is the number of weekly tests for influenza (most were likely panel tests). The baseline accounts for routine tests in the hypothetical absence of influenza, RVS, adenovirus and parainfluenza activity, and corresponds to the model estimate of the number of tests that were truly negative for all tested viruses. The blue area (light plus dark) corresponds to tests attributed to influenza, with the light blue area corresponding to tests confirmed positive for influenza. The purple area (light plus dark) corresponds to tests attributed to RSV, adenovirus or parainfluenza. The light purple area is the total number confirmed positive for these viruses.

The attribution of influenza negative test results to influenza and other viruses is illustrated in Figure 3. The baseline curve is the model estimate of the number of tests that were likely truly negative for all four viruses tested. A reduction in specimen collection and testing, primarily for viruses other than influenza, is also evident over the Christmas period (Figure 3).

The weekly proportion of tests confirmed positive for influenza peaked each season at 15 to 30%. Accounting for the model estimated false negative rate suggests that during periods of peak influenza activity, 40–90% of tests were performed on specimens taken from persons recently infected with influenza. Influenza was confirmed in only 14% of specimens sent for testing over the winter period, whereas the sensitivity estimate would imply that up to 40% of influenza tests could be attributed to an influenza infection. The corresponding figures for the whole year indicate that 10% of specimens were confirmed positive for influenza and 30% of influenza tests could be model-attributed to an influenza infection annually.

Despite a relatively large number of tests in the off-season, the number of influenza positive tests was almost negligible; suggesting that the false positive rate applicable to RVDSS influenza testing is minimal.

## Discussion

The model estimated sensitivity based on influenza test results reported to the RVDSS of 30–40% is much lower than the standard assay sensitivities documented in the literature. Standard sensitivities for diagnostic procedures used by participating laboratories ranged from 64% for rapid antigen tests to 95% for RT-PCR tests, averaging 75% for the study period [23]. As performance characteristics of specific tests are generally based on high quality specimens, the difference of approximately 40% is likely linked to any one of many operational procedures that affects the quality of the specimen and its procurement. Unlike validation studies, our samples are taken from a variety of clinical settings and processed with a variety of procedures across the country. As well, variation in the indications for diagnostic testing may vary across the country.

As there are many other respiratory pathogens that are not routinely tested for, or reported to the RVDSS, including human metapneumovirus (hMPV), coronaviruses, and rhinoviruses for which patients may seek medical care and present with influenza like illness [29]–[32], a large proportion of negative test results was expected. The overall model fit, and the general consistency of the sensitivity estimates, suggests that these many respiratory viruses were reasonably accounted for by the seasonal baseline and that the strong association between the number of influenza positive and influenza negative tests on a weekly basis is indicative of a significant number of false negative results, rather than the activity of another virus or viruses exactly synchronous with influenza. The latter would bias the estimated sensitivity of the system downwards. However, to significantly and consistently bias the estimate, the degree of synchronization would have to be fairly strong, persist over the whole study period, and occur in all provinces. Synchronization was not observed among the RVDSS viruses (influenza A, influenza B, RSV, adenovirus and PIV), and elsewhere other viruses such as rhinovirus, coronavirus and hMPV accounted for only a small proportion of the viral identifications and were not found to be synchronized with influenza [33]. As well, patients may present for care due to a secondary bacterial infection. While any specimen would likely test negative as the virus, at this point, is likely not detectable, the model would statistically attribute a negative test in this case to the primary infection; one of the four RVDSS viruses or to the seasonal baseline that represents other respiratory infections, depending on the level of viral activity at the time of the test. This is not considered a source of bias.

The large variation in false negative rates estimated for individual laboratories reporting to the RVDSS suggests that standardization of sample procurement, testing and reporting procedures would likely reduce the overall false negative rate. The accuracy of diagnostic tests is known to be affected by the quality of the specimen [10], [11], its handling, the timing of collection after symptom onset, and the age of the patient [14], [15]. Even with the most sensitive molecular methodologies, yield was shown to be strongly related to the time since onset of symptoms [9], [14], with a 3-fold decline in proportion positive within 3 to 5 days after onset of symptoms for both RT-PCR and culture procedures. For most laboratory tests, specimen procurement within 72 hours of from the onset of symptoms is recommended [6], yet patients often present much later in the course of illness. Estimates of the median time since onset of symptoms suggest a delay of 3 and 5 days for outpatient and inpatients respectively [15], however these estimates are limited to patients with laboratory confirmed influenza. In addition, there are inherent differences in the performance characteristics of the currently used diagnostic tests [4], [6], [8], [34]–[38]. Lack of standardization between diagnostic tests and algorithms used in different laboratories reporting to the RVDSS adds to this complexity. The routine use of RT-PCR testing has only recently become available in Canada (only 20% of tests used RT-PCR methods as of 2005/06 [23]), but increased use of this modality is expected to improve accuracy.

Population or system level sensitivity estimates that include the effects of sample quality are limited. Grijalva and colleagues [39] estimated the diagnostic sensitivity in a capture recapture study of children hospitalized for respiratory complications at 69% for a RT-PCR based system and 39% for a clinical-laboratory based system (passive surveillance of tests performed during clinical practice, and using a variety of commercially available tests).

Though the expected proportion of influenza tests that were due to influenza infections is unknown and variable, our model estimate of 30% appears plausible. Cooper and colleagues [33] attributed 22% of telephone health calls for cold/flu to influenza over two relatively mild years, and elsewhere 20% of admissions for acute respiratory infections (including influenza) in adults aged 20–64 years were attributed to influenza, and 42% for seniors [1].

While there are limitations with this approach, there are no other simple alternatives to assist in the interpretation of the RVDSS data. It would have been helpful to analyze data based on each specimen sent for testing. With only the number of weekly tests and number of positive results, we were unable to calculate the number of specimens that were actually found to be negative for all four viruses, or to estimate the extent of co-infection. Co-infection, which was not accounted for in our model, could result in an under-estimation of the number of falsely negative tests, as the attribution of an influenza negative test that was actually co-infected with influenza and another respiratory virus would have to be split between the viruses. With auxiliary information associated with each specimen, model estimates of false negative rates based on, for example, test type, time since onset of symptoms, age of the patient, or clinical presentation would have allowed us to explore the reasons for the high false negative rates. As the false negative rate appears to be laboratory dependant (data not shown), this estimated range is applicable only to the RVDSS for the study period. A significant reduction in the false negative rate is anticipated as methods become standardized and with the uptake of the new RT-PCR methods. As positive results, particularly for culture, are often obtained a week or more after the specimen was received, some positive results may have been reported in a different week than the test. Multiple test results for a single specimen may have also contributed to reporting irregularities. These irregularities would tend to bias the estimated parameter towards zero, and hence the estimated sensitivity towards 1. Considering the overall model fit and the relative severity of influenza [1], we conclude that our estimate of sensitivity may be slightly over-estimated (number of false negatives under-estimated).

Poor test sensitivity contributes to the chronic under-estimation of the burden of influenza in the general population. Since estimates of the burden of illness drive planning for preventive and therapeutic interventions, it is important to improve all aspects leading to improved diagnostic accuracy. We have illustrated a simple method that uses the surveillance data itself to estimate the system wide sensitivity associated with the weekly proportion of tests confirmed positive. Although our estimate of sensitivity is only applicable to the interpretation of the RVDSS data over the study period, similar estimates for specific cohorts or laboratory procedures may help guide further investigation into the reasons for the large number of false negative test results. The capacity for improved diagnostic accuracy will ultimately improve our understanding of the epidemiology of influenza.

## References

[1] Schanzer DL, Langley JM, Tam TWS (2008). Role of influenza and other respiratory viruses in admissions of adults to Canadian hospitals.. Influenza and Other Respiratory Viruses.

[2] Schanzer DL, Langley JM, Tam TWS (2008). Co-morbidities associated with influenza-attributed mortality, 1994–2000, Canada.. Vaccine.

[3] Schanzer DL, Tam TWS, Langley JM, Winchester BT (2007). Influenza-attributable deaths: Canada 1990-1999.. Epidemiol Infect.

[4] Weinberg A, Mettenbrink CJ, Ye D, Yang C-F (2005). Sensitivity of diagnostic tests for influenza varies with the circulating strains.. J Clin Virol.

[5] Grijalva CG, Poehling KA, Edwards KM, Weinberg GA, Staat MA (2007). Accuracy and Interpretation of Rapid Influenza Tests in Children.. Pediatrics.

[6] Petric M, Comanor L, Petti CA (2006). Role of the Laboratory in Diagnosis of Influenza during Seasonal Epidemics and Potential Pandemics.. J Infect Dis.

[7] Hatchette TF, Bastien N, Berry J, Booth TF, Chernesky M (2009). The limitations of point of care testing for pandemic influenza: what clinicians and public health professionals need to know.. Can J Public Health.

[8] Erdman DD, Weinberg GA, Edwards KM, Walker FJ, Anderson BC (2003). GeneScan Reverse Transcription-PCR Assay for Detection of Six Common Respiratory Viruses in Young Children Hospitalized with Acute Respiratory Illness.. J Clin Microbiol.

[9] Leitmeyer K, Buchholz U, Kramer M, Schweiger B (2002). Enhancing the predictive value of throat swabs in virological influenza surveillance.. Euro Surveill.

[10] Daley P, Castriciano S, Chernesky M, Smieja M (2006). Comparison of Flocked and Rayon Swabs for Collection of Respiratory Epithelial Cells from Uninfected Volunteers and Symptomatic Patients.. J Clin Microbiol.

[11] Robinson JL, Lee BE, Kothapalli S, Craig WR, Fox JD (2008). Use of Throat Swab or Saliva Specimens for Detection of Respiratory Viruses in Children.. Clin Infect Dis.

[12] Heikkinen T, Marttila J, Salmi AA, Ruuskanen O (2002). Nasal Swab versus Nasopharyngeal Aspirate for Isolation of Respiratory Viruses.. J Clin Microbiol.

[13] van de Pol AC, van Loon AM, Wolfs TFW, Jansen NJG, Nijhuis M (2007). Increased Detection of Respiratory Syncytial Virus, Influenza Viruses, Parainfluenza Viruses, and Adenoviruses with Real-Time PCR in Samples from Patients with Respiratory Symptoms.. J Clin Microbiol.

[14] Wallace LA, Collins TC, Douglas JDM, McIntyre S, Millar J (2004). Virological surveillance of influenza-like illness in the community using PCR and serology.. J Clin Virol.

[15] Steininger C, Kundi M, Aberle SW, Aberle JH, Popow-Kraupp T (2002). Effectiveness of Reverse Transcription-PCR, Virus Isolation, and Enzyme-Linked Immunosorbent Assay for Diagnosis of Influenza A Virus Infection in Different Age Groups.. J Clin Microbiol.

[16] Schanzer DL, Langley JM, Tam TWS (2006). Hospitalization attributable to influenza and other viral respiratory illnesses in Canadian children.. Pediatr Infect Dis J.

[17] Schanzer DL, Langley JM, Tam TWS (2007). Influenza-attributed hospitalization rates among pregnant women, 1994–2000, Canada.. J Obstet Gynaecol Can.

[18] Thompson WW, Shay DK, Weintraub E, Brammer L, Bridges CB (2004). Influenza-associated hospitalizations in the United States.. JAMA.

[19] Reyes F, Macey JF, Aziz S, Li Y, Watkins K (2007). Influenza in Canada: 2005–2006 season.. Can Commun Dis Rep.

[20] Public Health Agency of Canada (2008). FluWatch Reports.. http://www.phac-aspc.gc.ca/fluwatch/index-eng.php.

[21] Aziz S, Tam T, Macey J, Li Y, Jain S (2005). Influenza in Canada: 2003–2004 season.. Can Commun Dis Rep.

[22] McGeer A, Green KA, Plevneshi A, Shigayeva A, Siddiqi N (2007). Antiviral Therapy and Outcomes of Influenza Requiring Hospitalization in Ontario, Canada.. Clin Infect Dis.

[23] Garner M, Garner R, Macey J, Tam T, Aziz S, Smieja M (2008). Impact of Changing Laboratory Diagnostics on Influenza Surveillance..

[24] SAS Institute Inc (2002). SAS/STAT**® 9** User's Guide, Volumes 1, 2, 3.

[25] Arens MQ, Swierkosz EM, Schmidt RR, Armstrong T, Rivetna KA (1986). Strategy for efficient detection of respiratory viruses in pediatric clinical specimens.. Diagn Microbiol Infect Dis.

[26] Rachlis ML (2004). Prescription for Excellence: How Innovation is Saving Canada's Health Care System.

[27] Upfold J (2002). Emergency department overcrowding: ambulance diversion and the legal duty to care.. CMAJ.

[28] Bhat N, Wright JG, Broder KR, Murray EL, Greenberg ME (2005). Influenza-associated deaths among children in the United States, 2003–2004.. N Engl J Med.

[29] Louie JK, Hacker JK, Gonzales R, Mark J, Maselli JH (2005). Characterization of Viral Agents Causing Acute Respiratory Infection in a San Francisco University Medical Center Clinic during the Influenza Season.. Clin Infect Dis.

[30] Walsh EE, Peterson DR, Falsey AR (2008). Human metapneumovirus infections in adults: another piece of the puzzle.. Arch Intern Med.

[31] Falsey AR (2008). Human metapneumovirus infection in adults.. Pediatr Infect Dis J.

[32] Bastien N, Ward D, Van Caeseele P, Brandt K, Lee SH (2003). Human metapneumovirus infection in the Canadian population.. J Clin Microbiol.

[33] Cooper DL, Smith GE, Edmunds WJ, Joseph C, Gerard E (2007). The contribution of respiratory pathogens to the seasonality of NHS Direct calls.. J Infect.

[34] Weinberg GA, Erdman DD, Edwards KM, Hall CB, Walker FJ (2004). Superiority of reverse-transcription polymerase chain reaction to conventional viral culture in the diagnosis of acute respiratory tract infections in children.. J Infect Dis.

[35] Espy MJ, Uhl JR, Sloan LM, Buckwalter SP, Jones MF (2006). Real-Time PCR in Clinical Microbiology: Applications for Routine Laboratory Testing.. Clin Microbiol Rev.

[36] Weinberg A, Walker ML (2005). Evaluation of Three Immunoassay Kits for Rapid Detection of Influenza Virus A and B.. Clin Diagn Lab Immunol.

[37] Hurt AC, Alexander R, Hibbert J, Deed N, Barr IG (2007). Performance of six influenza rapid tests in detecting human influenza in clinical specimens.. J Clin Virol.

[38] Ruest A, Michaud S, Deslandes S, Frost EH (2003). Comparison of the Directigen Flu A+B Test, the QuickVue Influenza Test, and Clinical Case Definition to Viral Culture and Reverse Transcription-PCR for Rapid Diagnosis of Influenza Virus Infection.. J Clin Microbiol.

[39] Grijalva CG, Weinberg GA, Bennett NM, Staat MA, Craig AS (2007). Estimating the undetected burden of influenza hospitalizations in children.. Epidemiol Infect.

